# Na_2_B_6_Si_2_: A Prototype Silico-boride with Closo (B_6_)^2–^ Clusters

**DOI:** 10.1021/jacs.4c08745

**Published:** 2024-08-26

**Authors:** Wilder Carrillo-Cabrera, Julia-Maria Hübner, Riccardo Freccero, Walter Jung, Michael Baitinger, Juri Grin, Ulrich Schwarz

**Affiliations:** † Max-Planck-Institut für Chemische Physik fester Stoffe, Nöthnitzer Straße 40, 01187 Dresden, Germany; ‡ Earth and Planets Laboratory, Carnegie Institution for Science, Washington, District of Columbia 20015, United States; § Faculty of Chemistry and Food Chemistry, TUD Dresden University of Technology, 01062 Dresden, Germany; ∥ Dipartimento di Chimica e Chimica Industriale, Università degli Studi di Genova, Via Dodecaneso 31, I-16146 Genova, Italy

## Abstract

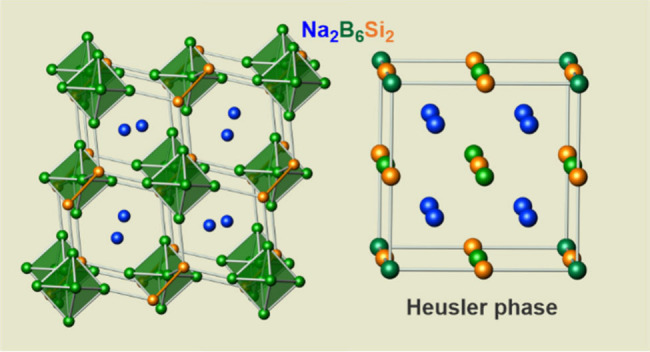

The compound Na_2_B_6_Si_2_ was synthesized under high-pressure, high-temperature conditions at pressures ranging from 6 to 9.5 GPa and temperatures from 1070 to 1270 K before quenching to room temperature followed by slow decompression. The crystal structure was determined from microcrystals using precession-assisted electron diffraction tomography, validated by dynamical refinement and full-profile refinements using optimized coordinates from quantum chemical calculations (space group *R*3̅*m*, Pearson symbol *hR*30, *a* = 5.0735(1) Å and *c* = 16.0004(7) Å). The atomic arrangement consists of a unique framework formed by electron-precise octahedral closo (B_6_)^2–^ clusters connected via ethane-like (Si_2_)^0^ dumbbells. The Na^+^ cations occupy cavities in the hierarchical variation of a Heusler-type framework. The balance (Na^+^)_2_([B_6_]^2–^)(Si^0^)_2_ reveals an electron precise Zintl-Wade phase, which is in line with electronic band structure calculations predicting semiconducting behavior.

The crystal chemistry of boron-rich intermetallic compounds is characterized by deltahedral boron clusters with specific electron counts.^1−4^ In silico-borides of high boron content, boron–silicon networks typically consist of icosahedral (*B*_12_)^2–^ closo clusters and tetrahedrally coordinated Si^0^ atoms, as observed, e.g., in the crystal structures of Na_8_B_74.5_Si_17.5_, YB_17.6_Si_4.6_, and Li_2_B_12_Si_2_.^5−7^ An analogous sodium phase Na_2_B_12_Si_2_ is predicted to be stable, and calculations indicate that the compound is a promising hard material.^8^ At high pressures, however, access is granted to the more uncommon structural arrangement of Na_2_B_6_Si_2_. The compound was discovered during high-pressure syntheses of Na_8_B_4_Si_42_ with a clathrate-VIII-type crystal structure.^9^ In the following experiments, it turned out that Na_2_B_6_Si_2_ could not be made from stoichiometric amounts of elemental components. The BN crucibles used for the synthesis proved to be not inert against the reactive mixture under high-pressure high-temperature conditions, and the sodium content in the product decreased with reaction time. Therefore, an excess of sodium was required and the reactants NaSi and amorphous boron were used in a ratio of 5:2.^10−12^ Na_2_B_6_Si_2_ forms in samples prepared at pressures ranging from 6 to 9.5 GPa. After a short 10 min reaction at *p* = 6 GPa and *T* = 1220(100) K, powder X-ray diffraction data revealed approximately 15 at-% Na_2_B_6_Si_2_, and 85 at-% clathrate-VIII phase. After prolonging the annealing time to 180 min, the sodium had reacted with the crucible material, and only (*cF*8)Si reflections were detected. The highest yield of about 30 atom % Na_2_B_6_Si_2_ was obtained after 1 h of reaction time at *p* = 8 GPa and *T* = 1270(100) K before quenching to room temperature and subsequent slow decompression. Powder X-ray diffraction patterns of the product showed reflections of BN and silicon, indicating crucible decomposition. In the inert atmosphere of a glovebox, the product transforms within several months into an amorphous product pointing at its metastable nature. When exposed to air and moisture, the target product remains stable for at least weeks.

To elucidate the crystal structure of Na_2_B_6_Si_2_, thin lamellar samples were cut from grains of the 6 GPa specimen by using the focused ion beam method and isolated by the lift-out technique. Scanning electron microscopy images revealed tiny grains of Na_2_B_6_Si_2_ precluding single-crystal X-ray diffraction experiments (Figure S1). Thus, selected area electron diffraction images were collected, revealing the trigonal symmetry of the Na_2_B_6_Si_2_ crystal structure, along with its approximate unit cell parameters (Figures 1 and S2). The reflection conditions −*h* + *k* + *l* = 3*n* for *hkl* and *l* = 3*n* for 00*l* were compatible with space groups *R*3, *R*3̅, *R*32, *R*3*m*, and *R*3̅*m*. Precession electron diffraction and tomography data collection resulted in 474 symmetry-independent reflections with *I* > 2σ(*I*) (Figures 1 and S3, Table S1). The crystal structure was first refined with a kinematical software using the precession electron diffraction data to a residual value *R*_F_ of 0.21.^13,14^ For a subsequent dynamical refinement, 1287 symmetry-independent reflections with *I* > 2σ(*I*) were used and resulted in *R*_F_ = 0.097. The derived composition Na_2_B_6_Si_2_ of the structure model (Table S2) is consistent with that of Na_1.8_B_5.9_Si_2.3_ determined by EDXS analysis.

**Figure 1:**
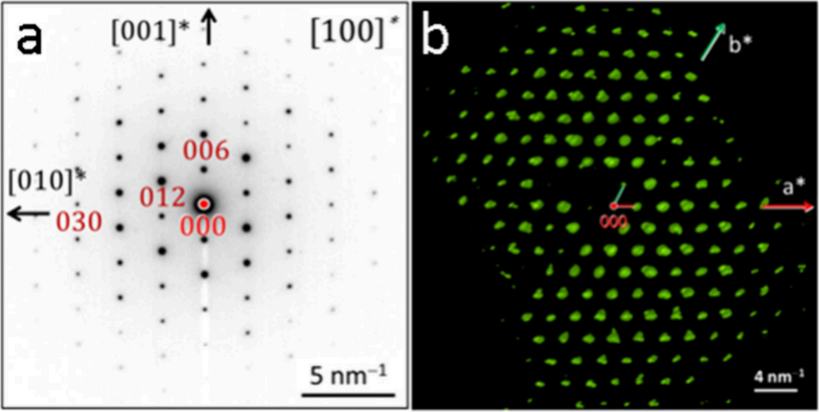
(a) Indexed SAED diffraction images of Na_2_B_6_Si_2_ taken along [100]*. (b) Projection of the electron diffraction volume along [001]*.

In addition, the starting model was refined with powder X-ray diffraction data (Table S1).^15^ Because of the byproducts in the powder specimen and the small scattering factor of boron, refinement of the atomic positions of Na_2_B_6_Si_2_ (space group *R*3̅*m*, *a* = 5.0735(1) Å, *c* = 16.0004(7) Å) yielded similar residuals for different parameter value combinations. Thus, the atomic positions were optimized using quantum chemical calculations with the experimental lattice parameters (Table S3) in analogy to an earlier established procedure.^16^ Those values were used as input for a final refinement using full diffraction profiles (Figures S4 and S5). The crystal structure determined (Table 1) represents a new structure type.

**Table 1:** Positional and Displacement Parameters of Na_2_B_6_Si_2_ Obtained from Rietveld Refinement Using the Starting Model of the Electron Diffraction Data Improved by Quantum Chemical Optimization of the Coordinates (Space Group *R*3̅*m* with *a* = 5.0735(1) Å and *c* = 16.0004(7) Å)

Atom	Site	*a*/*x*	*b*/*y*	*c*/*z*	*U*_*iso*_*/pm*^2^
Na	6*c*	0	0	0.2794(2)	0.054(2)
B	18*h*	0.2178(4)	2*x*	0.1224(3)	0.032(2)
Si	6*c*	0	0	0.0745(1)	0.036(1)

The atomic arrangement contains four-bonded (4*b*) silicon atoms grouped into (Si_2_)^0^ dumbbells and closo (B_6_)^2–^ clusters with six exobonds to silicon (Figure 2a). The structure may be seen as a hierarchical variation of Heusler phase Cu_2_MnAl, with the Al atoms substituted by B_6_ polyhedrons, the Mn atoms by Si_2_ dumbbells, and the Cu atoms by Na atoms (Figure S6). The occurrence of closo (B_6_)^2–^ clusters in silico-borides is observed for the first time. Under pressure, they replace the (B_12_)^2–^ clusters, which occur in the structurally related compounds Li_2_B_12_Si_2_ and MgB_12_Si_2_.^7,17^ The similarity between Na_2_B_6_Si_2_ and Li_2_B_12_Si_2_, however, is not limited to their obviously similar electron balance. By cutting the Si_2_ dumbbells, the 3D network of the Na_2_B_6_Si_2_ structure can be formally divided into layers perpendicular to the [001] direction (Figure 2b). In both structures, the layers are characterized by puckered six-membered rings embedding closo Wade clusters (Figure S7). In Li_2_B_12_Si_2_ the layers contain closo (B_12_)^2–^, and in Na_2_B_6_Si_2_ closo (B_6_)^2–^ anions. Such a crystallographic feature has been discussed for the structurally related gallides Na_2_Ga_7_ and NaLiGa_7_.^16,18,19^ The sodium atoms in Na_2_B_6_Si_2_ are situated in cavities of the B–Si framework (Figure 2c). The contacts *d*(Si–Si) of 2.384 Å are similar to that in (*cF*8)Si, and the endohedral distances of 1.742 and 1.758 Å (Table S4) fall in the normal range of (B_6_)^2–^ octahedra in binary *M*B_6_ compounds.^20−23^

**Figure 2:**
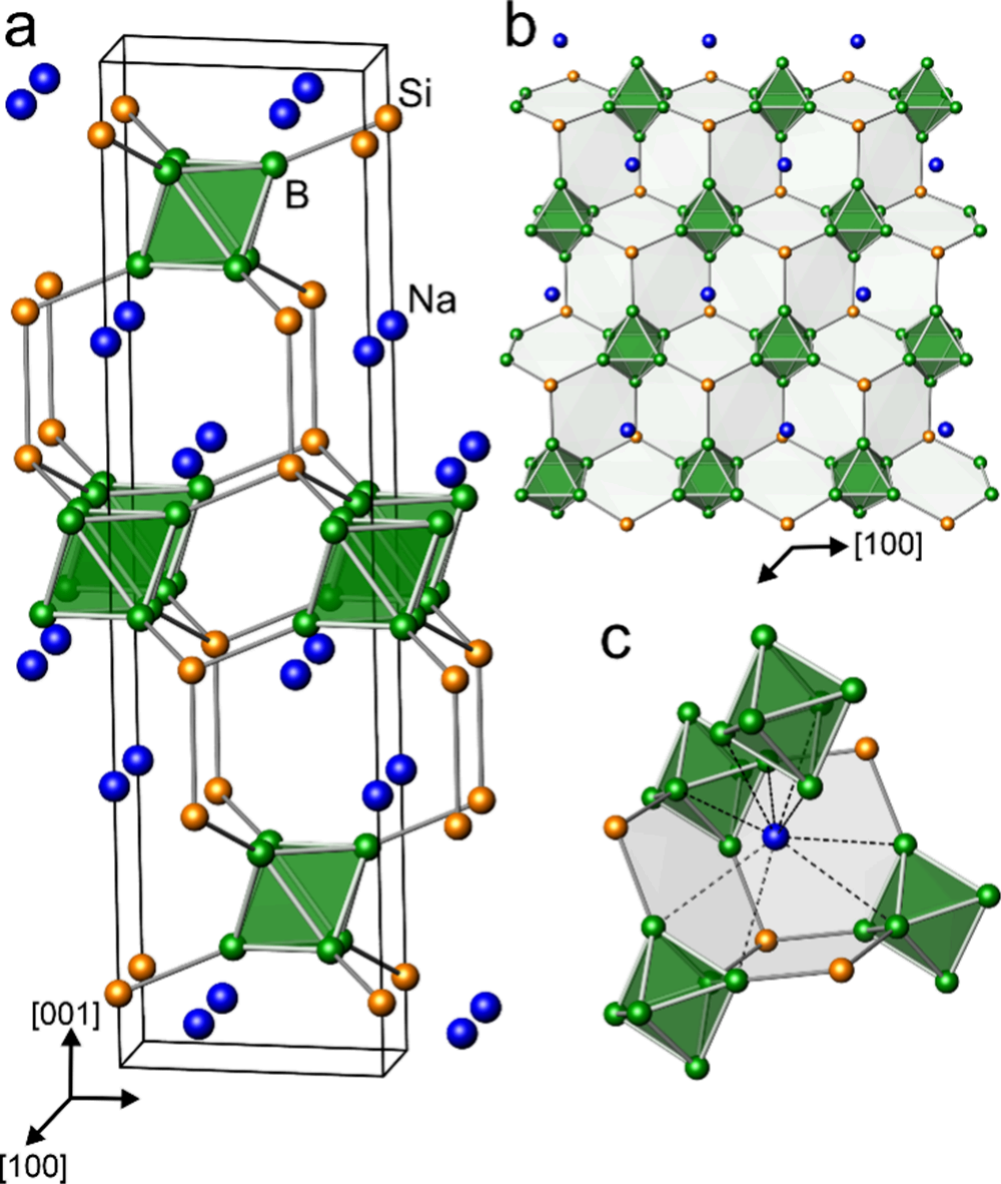
(a) Crystal structure of Na_2_B_6_Si_2_. (b) Layers perpendicular [001]. (c) Local environment of sodium. Boron atoms are depicted in green, silicon atoms in orange, and sodium atoms in blue.

For calculations of the bulk modulus, full crystal structure relaxations as a function of pressure were performed with the all-electron FHI-aims DFT-software package using the PBEsol exchange-correlation functional.^24−27^ The FINDSYM utility was used to determine the symmetry of the relaxed structures.^28^ The selected Brillouin zone sampling of 12 × 12 × 4 was selected after systematic testing for total energy convergence with respect to the number of *k*-points (Table S5). Excellent fitting of the calculated total energies and volumes were obtained with a third-order Birch–Murnaghan isothermal equation of state (Figure S8). The resulting bulk modulus *B*_0_ and equilibrium volume *V*_0_ amount to 111.3 GPa and 351.90 Å^3^, respectively. For comparison, the corresponding *B*_0_ values of NaB_6_ and KB_6_ with closo-clusters (B_6_)^1–^ connected by B–B bonds amount to about 133 GPa, while those of the alkaline-earth hexaborides with connected (B_6_)^2–^ units range from 144 to 159 GPa.^29−34^ Thus, the calculated compressibility reveals that Na_2_B_6_Si_2_ is expected to be a moderately hard material.

Concerning electronic properties, the density of states shows a band gap of approximately 2 eV indicating semiconducting behavior (Figure 3) and an electron-balanced composition as computed with the FPLO code.^35^

**Figure 3:**
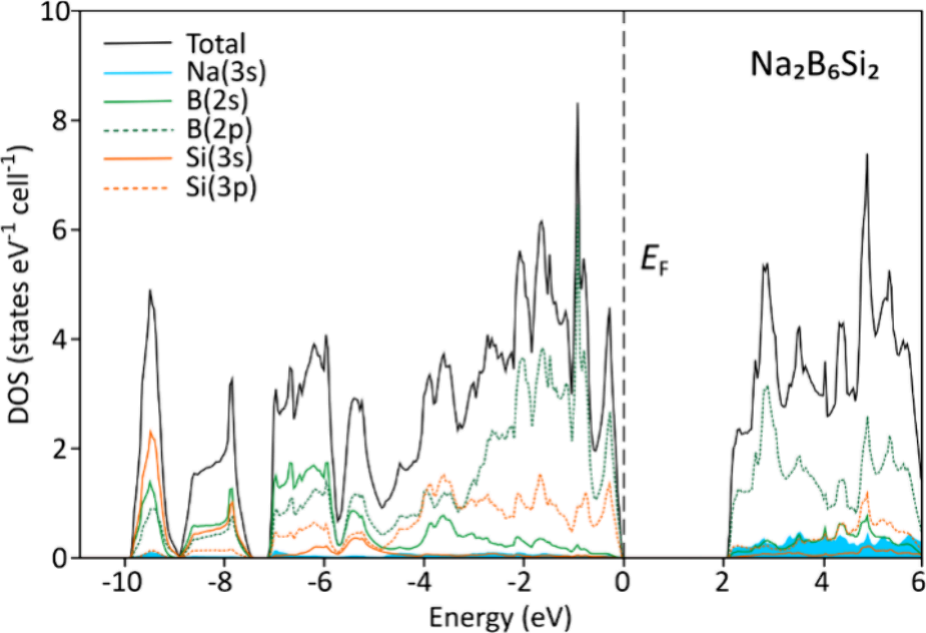
Electronic density of states of Na_2_B_6_Si_2_.

The chemical bonding in Na_2_B_6_Si_2_ was analyzed applying the electron localizability approach—a quantum chemical technique in position space.^36^ First, the effective charges were evaluated from the calculated electron density by using the QTAIM formalism.^37^ The zero–flux surfaces of the gradient vector field are defined as boundaries of basins, representing atomic regions (Figure S9). The obtained charge values of +0.83 for Na, +0.81 for Si, and −0.55 for B reveal a notable positive charge for silicon, in agreement with the Pauling scale (*EN*(Na) = 0.93, *EN*(B) = 2.04, *EN*(Si) = 1.90), and conceptual values from combined Zintl and Wade electron-counting schemes. Further information about the interactions between atoms is obtained from the common analysis of electron density and electron localizability indicator in its ELI-D representation.^36^ While the distribution of ELI-D in the regions of the inner shells is virtually spherical, the valence region is clearly structured, signaling the participation of these electrons in the bonding events. Only four types of ELI-D attractors (maxima) are found here, which visualize different bonds (Figure 4a and 4b). Three kinds of homoatomic B–B and Si–Si bonds are effectively two-atomic, although they have some minor contributions of sodium (or boron) ligands (less than 5% of the bond populations). In accordance with the conceptual picture, the population of the Si–Si bond (1.97 e) is close to 2, and the B–B bonds within the octahedral boron cluster are electron depleted (0.84 and 1.22 e, respectively, Figure 4). The B–Si bond (2.13 electrons) is rather polar: boron contributes 1.42 electrons, and silicon only 0.71 electrons. Such electron redistribution is the reason for the large QTAIM charge of silicon.

**Figure 4:**
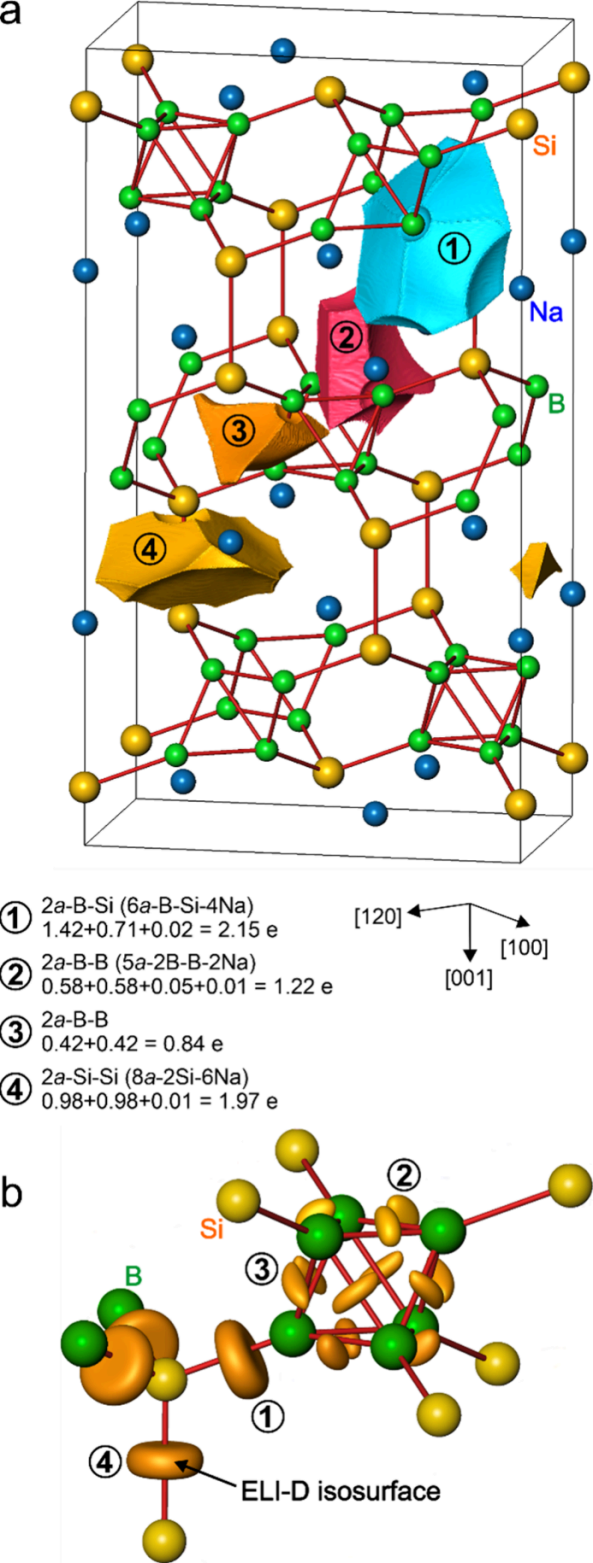
(a) ELI-D bonding basins. (b) ELI-D isosurfaces indicating covalent bonds in Na_2_B_6_Si_2_.

As a result of this study, we present a new structural motif for boron–silicon networks based on anionic B_6_ octahedra and four connected silicon atoms forming dumbbells. In accordance with the calculated band gap, Na_2_B_6_Si_2_ is an electron-precise valence compound with the balance (Na^+^)_2_[(B_6_)^2–^](Si^0^)_2_. While this structural motif can currently only be realized under extreme conditions, it offers a promising perspective for the development of new silico-borides that hold potential as lightweight materials with high stability.

## Supporting Information Available

Additional information on preparation, crystal structure solution by electron diffraction data, Rietveld refinements with optimized atomic positions, structural relationships of Na_2_B_6_Si_2_ as well as crystallographic data are supplied. (PDF)
