# Supplementary material for: Na2B6Si2: A Prototype Silico-boride with Closo (B6)2– Clusters
Source: J Am Chem Soc. 2024 Aug 26;146(36):24759–63. doi: 10.1021/jacs.4c08745 (PMC11403601; doi:10.1021/jacs.4c08745)
Supplement: Supplementary file 1 — ja4c08745_si_001.pdf [file ja4c08745_si_001.pdf]

01 Supporting information:

$Na_2B_6Si_2$: A prototype silico-boride with *closo* $[B_6]^{2-}$ clusters

Wilder Carrillo-Cabrera[1], Julia-Maria Hübner[1,2,3], Riccardo Freccero[4], Walter Jung[1], Michael Baitinger[1], Juri Grin[1], Ulrich Schwarz[1*]

[1]*Max-Planck-Institut für Chemische Physik fester Stoffe, Nöthnitzer Straße 40, 01187 Dresden, Germany.*

[2]*Earth and Planets Laboratory, Carnegie Institution for Science, Washington, District of Columbia 20015, United States*

[3]*Faculty of Chemistry and Food Chemistry, Technische Universität Dresden, 01062 Dresden, Germany*

[4] *Dipartimento di Chimica e Chimica Industriale, Università degli Studi di Genova, Via Dodecaneso 31, I-16146 Genova, Italy*

Table of Content:

1. Preparation
2. Crystal structure solution of $Na_2B_6Si_2$ from electron diffraction data
3. Rietveld refinement with the optimized atomic positions
4. Structural relationships
5. Calculated equation of state
6. QTAIM charges and bonding basins
7. Crystallographic data
8. *k*-point sampling
9. References

1. Preparation

Sample manipulations were conducted in argon-filled glove boxes. For high-pressure experiments, the precursor phases Na_4Si_4 and amorphous boron were used. Na_4Si_4 was synthesized from silicon (Chempur, 99.9999%) and sodium (Chempur, 99.95%) by annealing the mixture at 750 °C for 7 h in closed tantalum tubes, followed by slow cooling to room temperature over 8 h. Amorphous boron (Alfa Aesar) was purified and activated using a hydrogen plasma stream.[1]

A precursor mixture with a molar ratio of Na:B:Si = 5:2:5 was ground in an agate mortar and then transferred to BN crucibles (3 mm diameter), which were subsequently placed in MgO octahedrons (14 or 18 mm edge length). The high-pressure, high-temperature syntheses were carried out in a multi-anvil press with a Walker-type module.[2] Pressure and temperature were calibrated before the experiments by resistance measurements of elemental bismuth and thermocouple-calibrated runs, respectively. The samples were quenched under load. Post reaction, the products were cleansed with ethanol and deionized water to remove traces of Na_4Si_4, followed by additional washes with ethanol and acetone. Subsequently, the samples were dried at room temperature. The preparation conditions are detailed in Table S1.

Table S1. Preparation conditions of the high-pressure-high temperature syntheses and reaction products identified by PXRD.

Sample	Synthesis parameters	Clathrate I	Clathrate VIII	$Na_2B_6Si_2$	other phases
	5 GPa 1270 K 1 h	23%	77%	–	–
1a	6 GPa 1220 K 10 min	–	85 %	15 %	Traces of clathrate II identified by TEM
	6 GPa 1220 K 3 h	–	–	–	α-Si
1b	8 GPa 1270 K 1h	–	70 %	30 %	Si: 2 %<br>BN:16 %

2. Crystal Structure elucidation of $Na_2B_6Si_2$ from electron diffraction data

The crystal structure of $Na_2B_6Si_2$ was determined from a specimen isolated from the sample prepared at 6 GPa and 10 min annealing at 950 °C (Table S1). A specimen suitable for TEM investigations was prepared using the focused ion beam technique (FIB) with a Quanta 200 3D ion/electron dual-beam device (FEI, Eindhoven) equipped with an omniprobe micro-manipulator (W needle). Scanning electron microscopy (SEM) images revealed cubic shaped crystallites, which were utilized for subsequent investigations. Before sample cutting, protective Pt layers (24 µm long, 2 µm thick, 2 µm high) were deposited on the selected region using an acceleration voltage of 30 kV and a current of 0.1 nA. The cross-section with a thickness of 2 µm was prepared using a Ga-ion beam, operated at an acceleration voltage of 30 kV and a current ranging from 1 to 0.5 nA. The cut specimen was transferred onto the TEM holder using the in-situ lift-out technique.[3] Following this, the cross-section was thinned to 60 nm by using an acceleration voltage of 30 kV and currents of 0.5–0.01 nA of the Ga-ion beam. The TEM image of the lamella revealed crystalline areas (Figure S1) later identified as $Na_2B_6Si_2$ in addition to areas of the clathrate VIII phase $Na_8B_4Si_{42}$.[4]

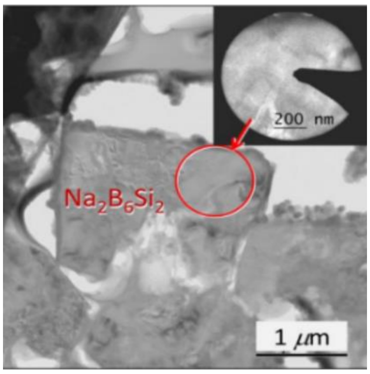

Figure S1. TEM images of FIB-lamella, showing crystals of $Na_2B_6Si_2$. The area chosen for the electron diffraction tomography experiment is marked. The inset shows the SAED aperture hole (840 nm diameter) and the top of the electron-beam stopper (black shadow).

Subsequent investigations were performed on FEI Tecnai F30-G2 super-twin microscope operating at 300 kV. The microscope was equipped with a CCD camera (GATAN Inc.) and a standard double-tilt holder (GATAN Inc.) with a tilting range of ± 46° of the holder axis and ± 30° perpendicular to it. The SAED mode was used for tomography data collection. Precession electron diffraction (PED) was performed using a DigiStar P1000 device (Nanomegas) and the images were processed and analyzed with Digital Micrograph (version 3.21.1374.0; GATAN Inc.) .

Selected area electron diffraction images revealed trigonal symmetry of the $Na_2B_6Si_2$ crystal structure, along with its approximate unit cell parameters a ≈ 5.03 Å and c ≈ 15.95 Å (Figure S2). The reflection conditions -*h*+*k*+*l* = 3*n* for *hkl* and *l* = 3*n* for 00*l* are compatible with the space groups *R*3, $R\bar{3}$, *R*32, *R*3*m*, and $R\bar{3}m$.

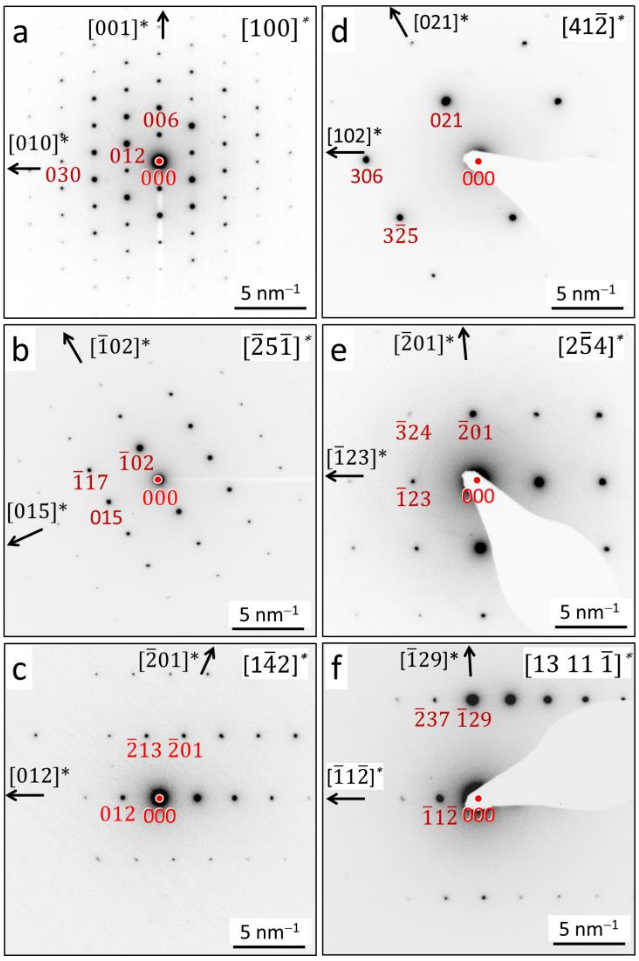

Figure S2. Indexed SAED diffraction images of $Na_2B_6Si_2$ taken along (a) [100]*, (b) [$\bar{2}$5$\bar{1}$]*, (c) [1$\bar{4}$2]*, (d) [41$\bar{2}$]*, (e), [2$\bar{5}$4]* and (f) [13 11 $\bar{1}$]* zone directions. The reflection conditions -*h*+*k*+*l* = 3*n* for *hkl* and *l* = 3*n* for 00*l* are compatible with the space groups *R*3, $R\bar{3}$, *R*32, *R*3*m*, and $R\bar{3}m$.

Precession electron diffraction (PED) was performed in SAED mode for tomography data collection. Crystal structure refinement in space group $R\overline{3}m$ converged to a residual value of R_g = 0.21 (474 reflections with $I > 2{\cdot}\sigma(I)$), for the dynamical refinement with 1287 reflections to R_d = 0.097. The refined composition $Na_2B_6Si_2$ agrees with the average composition determined by EDXS analysis of $Na_{18}B_{59}Si_{23}$.

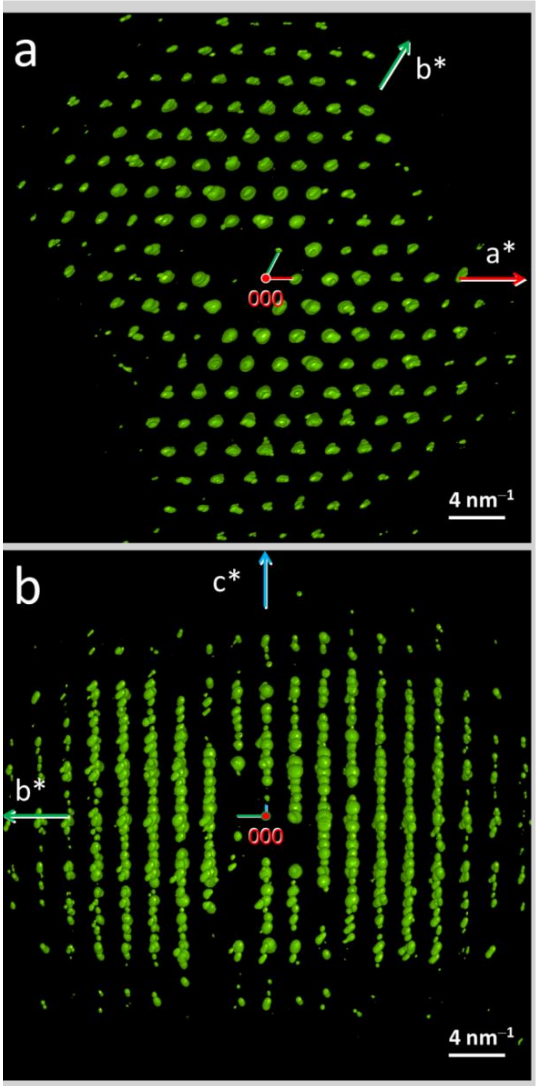

Figure S3. Projections of the electron diffraction volume for $Na_2B_6Si_2$ (a) along [001]* and (b) along [100]* zone directions, obtained by PED tomography.

3. Powder X-ray diffraction data refinement using full profiles and optimized coordinates of quantum-chemical computations.

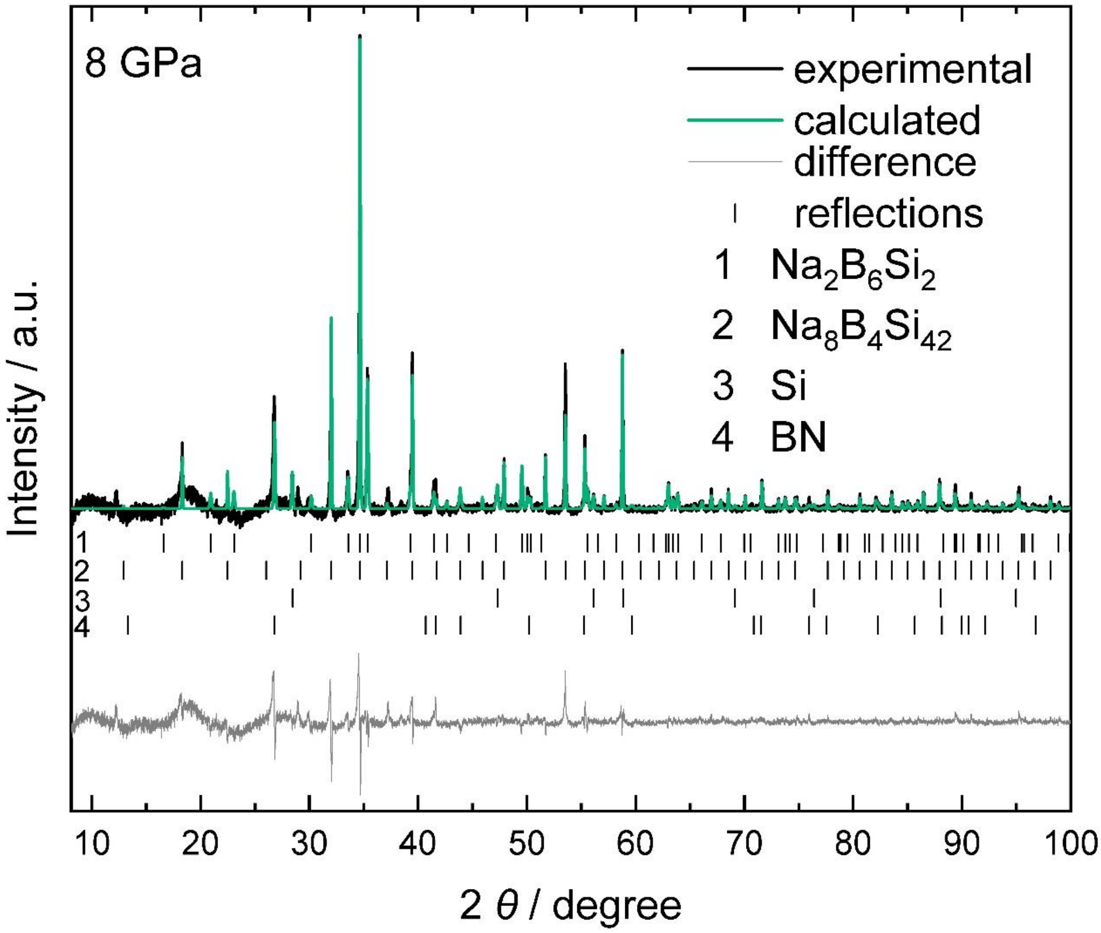

Figure S4. Powder X-ray diffraction pattern (background subtracted; Guinier camera, CuKα_1) with the residuals based on calculated profiles of the phases $Na_2B_6Si_2$, clathrate-VIII $Na_4B_4Si_{42}$, (*cF*8)Si and *h*-BN.

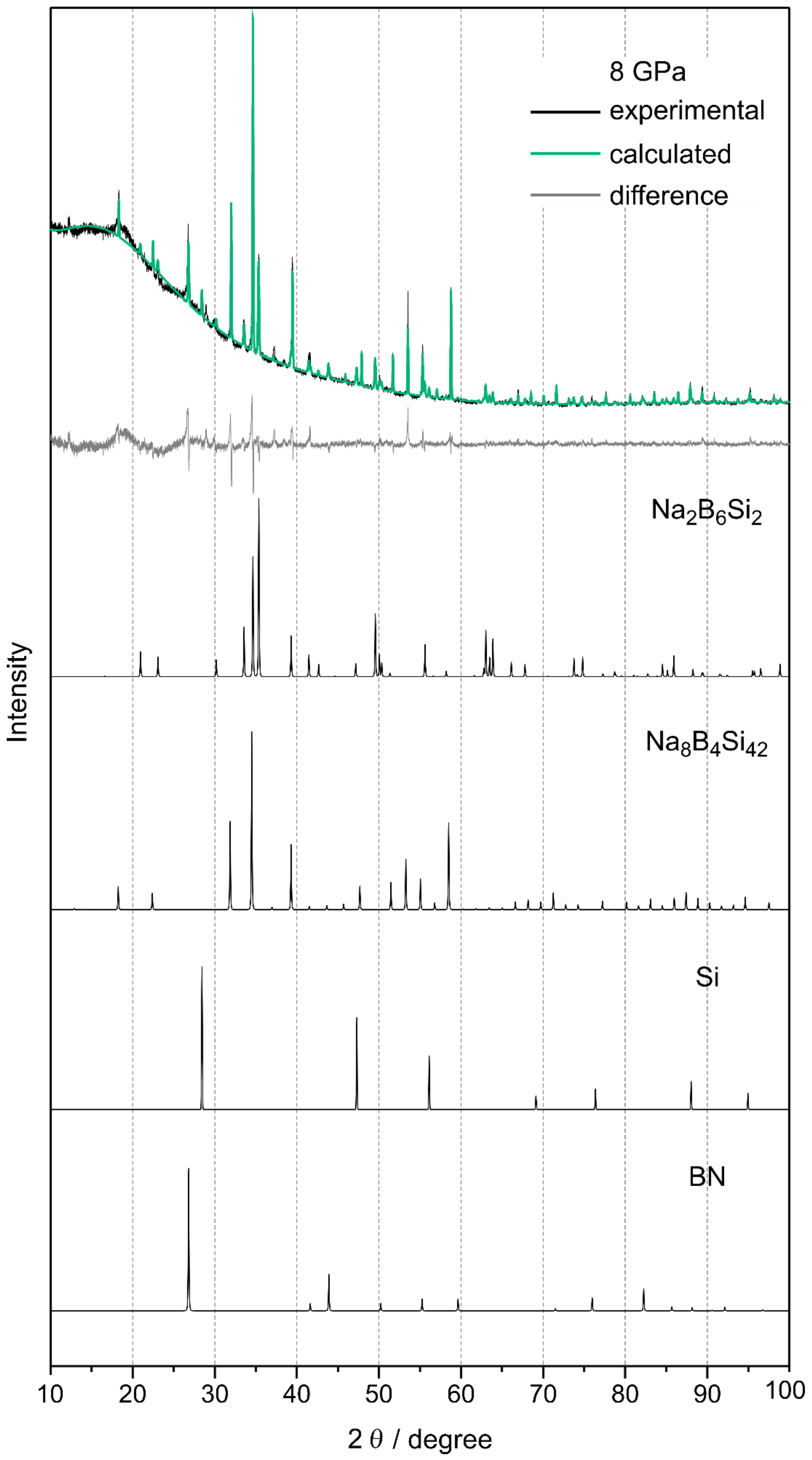

Figure S5. Powder X-ray diffraction pattern (raw data; Guinier camera, CuKα_1) and residuals. The calculated profiles are those of the phases $Na_2B_6Si_2$, clathrate-VIII $Na_4B_4Si_{42}$, (*cF*8)Si and hexagonal boron nitride.

4. Structural relationships

The crystal structure of $Na_2B_6Si_2$ constitutes a hierarchical variety of the Heusler phase Cu_2MnAl, in which the aluminum and manganese atoms are replaced by B_6 polyhedrons and Si_2 dumbbells, respectively.

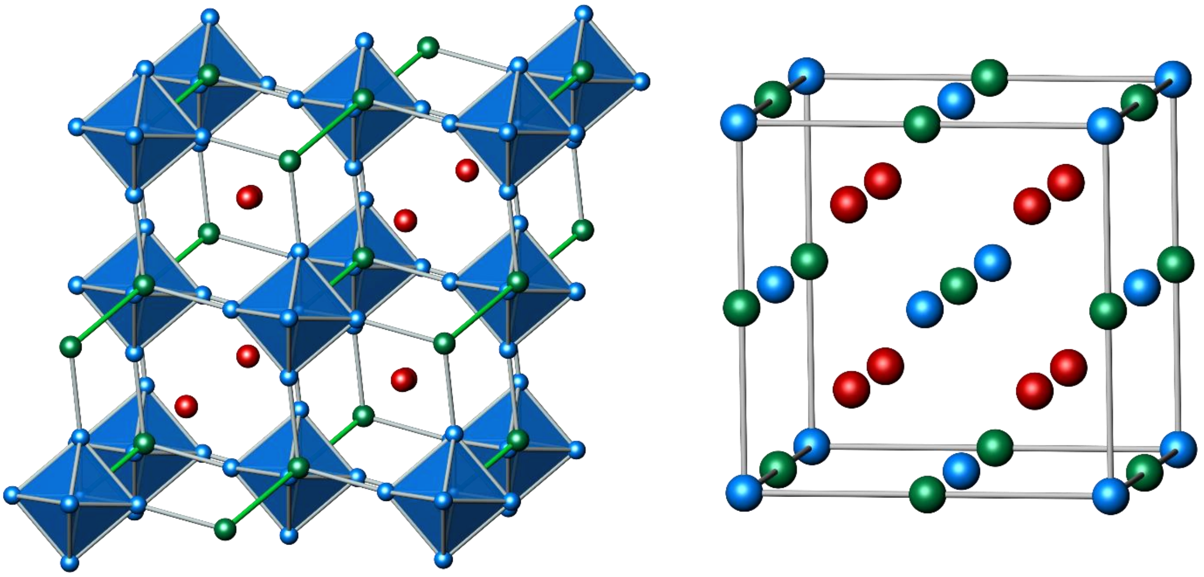

Figure S6. Relation between $Na_2B_6Si_2$ (left) and the Heusler phase Cu_2MnAl (right)

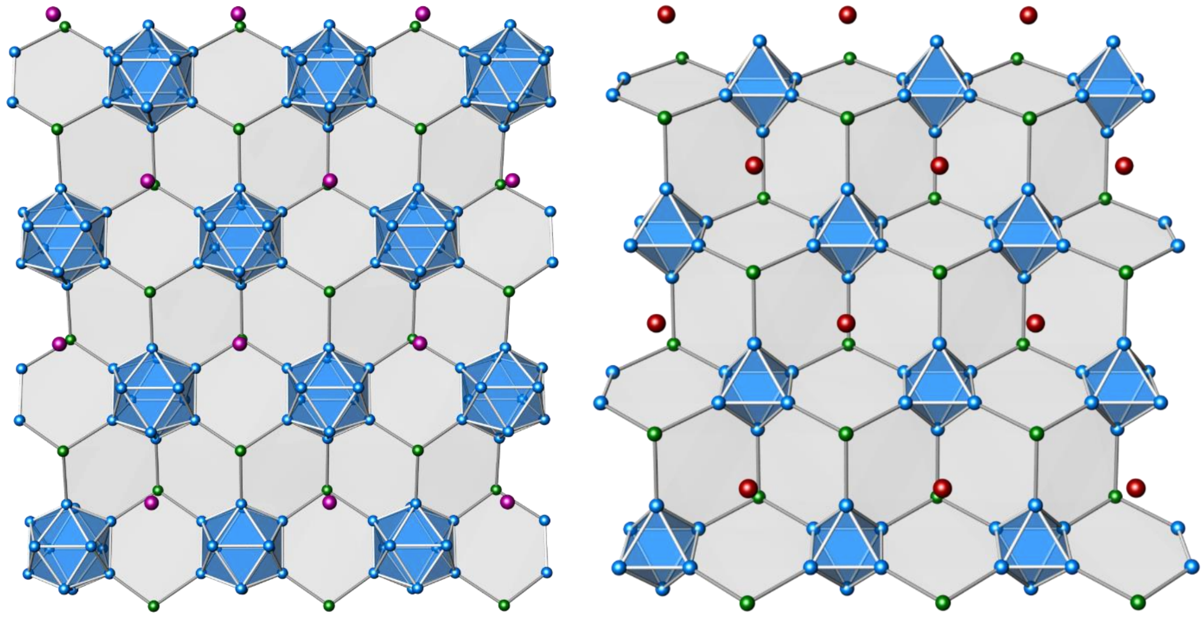

Figure S7. Layers in the crystal structure of $Li_2B_{12}Si_2$ (left) and $Na_2B_6Si_2$ (right). View perpendicular to [001].

5. Calculated equation of state

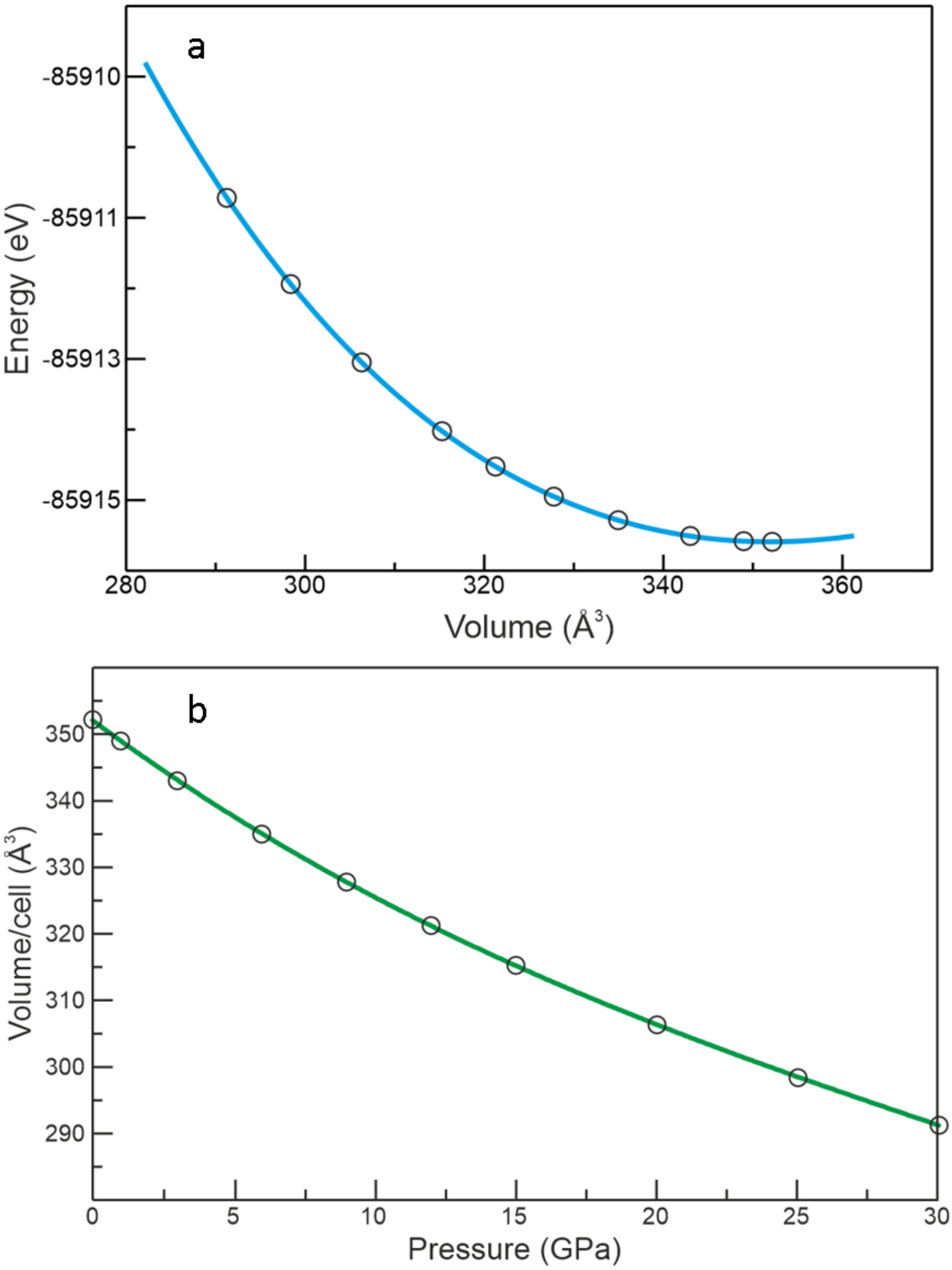

Figure S8. Calculated equation of state for $Na_2B_6Si_2$. a) Energy versus volume plot. The relaxed crystal structures retain the $R\bar{3}m$ symmetry up to 30 GPa. Excellent fitting of the calculated total energies and volumes were obtained with a 3rd-order Birch–Murnaghan isothermal equation of state. The resulting bulk modulus (B_0) and equilibrium volume (V_0) are 111.3 GPa and 351.90 Å^3, respectively. b) Equation of state in the form volume versus pressure with indicated least-squares fit to the data.

6. QTAIM shapes and bonding basins

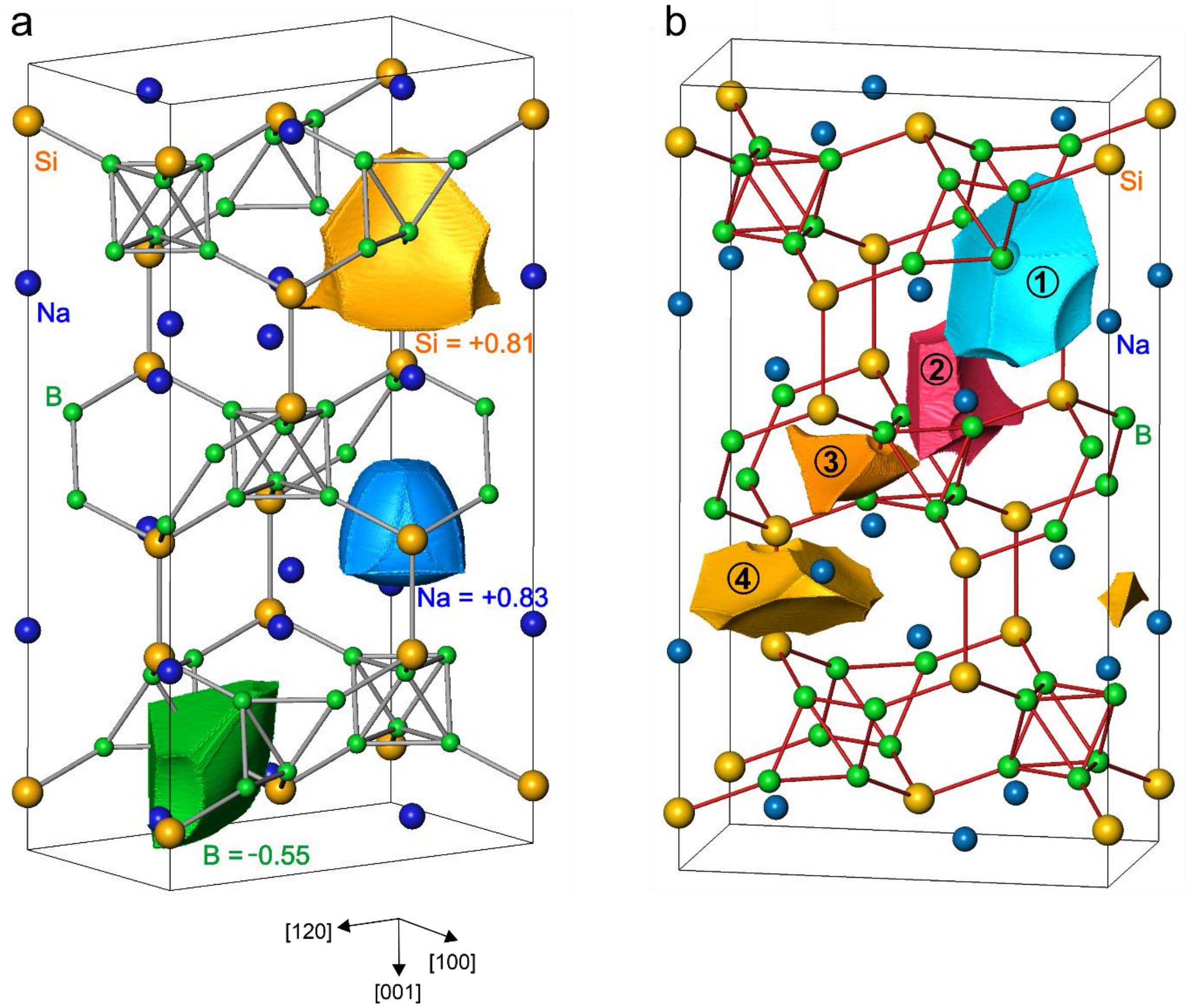

① 2*a*-B-Si (6*a*-B-Si-4Na)
1.42+0.71+0.02 = 2.15 e

② 2*a*-B-B (5*a*-2B-B-2Na)
0.58+0.58+0.05+0.01 = 1.22 e

③ 2*a*-B-B
0.42+0.42 = 0.84 e

④ 2*a*-Si-Si (8*a*-2Si-6Na)
0.98+0.98+0.01 = 1.97 e

Figure S9. a) QTAIM atomic shapes and charges and b) bond basins in the crystal structure of $Na_2B_6Si_2$.

7. Crystallographic data

Table S2. Data collection, structure refinement and crystallographic information for $Na_2B_6Si_2$.

Method	Single crystal electron diffraction	Powder X-ray diffraction
Synthesis	6 GPa, 950 °C, 30 min	8 GPa,1000 °C, 60 min
Formula, Z<br>Space group,<br>Pearson symbol	$Na_2B_6Si_2$; 3<br>$R\bar{3}m$ (no. 166),<br>*hR*30	
Unit-cell dimensions from PXRD	*a* = 5.0711(9) Å<br>*c* = 15.999(6) Å<br>*V* = 356.3(2) $Å^3$	*a* = 5.0735(1) Å,<br>*c* = 16.0004(7) Å;<br>*V* = 356.8(2) $Å^3$
Data collection	Tecnai F30-G[2] super-twin electron microscope (FEI)	Huber Image Plate Guinier Camera G670
Radiation	Electrons; λ = 0.01969 Å	CuKα_1; λ =1.54056 Å
2θ range	0.30° - 3.10°	10.00° - 100.40°
Data points Number	-	18081
Reflections Number, total / independent	17794 / 1306	60 / 60
$I > 2\sigma(I)$	1287	36
Absorption	Uncorrected	-
Dynamical effects	Corrected	-
Analyzing software	PETS2.0[5]<br>Jana2020[6]	Jana2006[7]<br>WinCSD[8]
Refined parameters	94	28
Largest diff. peak; hole	+0.56 e·$Å^{-3}$; –0.69 e·$Å^{-3}$	+1.21 e·$Å^{-3}$; –0.71 e·$Å^{-3}$
R_I dyn (cin)	0.097 (0.214)	-
R_p, R_{wp}	-	0.078, 0.085
R_{p-all}, R_{wp-all}	-	0.083, 0.086
Goodness of fit on F^2	2.42	1.64

Table S3. Atomic coordinates and isotropic displacement parameters for $Na_2B_6Si_2$ obtained by dynamical refinement of single-crystal electron diffraction data.

Atom	site	*x/a*	*y/b*	*z/c*	U_{iso}/Å^2
Na	6*c*	0	0	0.2789(1)	0.0132(5)
B	18*h*	0.2164(2)	2*x*	0.1222(1)	0.0039(5)
Si	6*c*	0	0	0.07451(9)	0.0037(4)

Table S4. Atomic coordinates and isotropic displacement parameters for $Na_2B_6Si_2$ as determined by quantum chemical optimization using experimental lattice parameters.

Atom	site	*x/a*	*y/b*	*z/c*
Na	6*c*	0	0	0.2794
B	18*h*	0.2178	2*x*	0.1224
Si	6*c*	0	0	0.0745

Table S5. Selected interatomic distances [Å] for $Na_2B_6Si_2$.

**Electrons Sample 1a**				**Optimized model**			
Si	– Si	2.377(4)		Si	– Si	2.384	
	– B	2.054(4)	3×		– B	2.062	3×
B	– Si	2.054(4)		B	– Si	2.062	
	– B	1.764(5)	2×		– B	1.742	2×
	– B	1.765(6)	2×		– B	1.758	2×
Na	– Si	2.945(2)	3×	Na	–Si	2.947	3×
	– Si	3.261(11)			– Si	3.278	
	– Si	3.587(6)	3×		– Si	3.579	3×
	– B	2.789(4)	6×		– B	2.799	6×
	– B	2.996(7)	3×		– B	3.000	3×
	– B	3.158(6)	3×		– B	3.158	3×
	–Na	3.408(4)	3×		– Na	3.401	3×

8. *k*-point sampling

Table S6. The convergence of the total energy with respect to the *k*-points sampling for $Na_2B_6Si_2$.The default width of 0.01 eV for the Gaussian broadening was used together with scalar-relativistic effects within the ZORA approximation.

Number of *k*-points (reduced by symmetry)	*k*-point mesh	Total energy (eV)
40	6x6x2	-85915.590858063
122	9x9x3	-85915.591119655
292	12x12x4	-85915.591118192
642	16x16x5	-85915.591118142
1084	19x19x6	-85915.591118144

9. References

[1] Alekseeva, A.; Kovnir, K.; Chizhov, P.; Baitinger, M.; Grin, Yu. Materials purification by treatment with hydrogen-based plasma. Eur. Pat. EP1893320 B8, **2010**.

[2] Walker, D.; Carpenter, M. A.; Hitch, C. M. Some simplifications to multianvil devices for high pressure experiments. *Am. Mineral.* **1990**, *75*, 1020– 1028.

[3] Fan, J.; Carrillo-Cabrera, W.; Akselrud, L.; Prots, Y.; Antonyshyn, I.; Chen, L.; Grin, Yu. New Monoclinic Phase at the Composition Cu2SnSe3 and Its Thermoelectric Properties. *Inorg. Chem.* **2013**, *52*, 11067–11074.

[4] Hübner, J.-M.; Carrillo-Cabrera, W.; Kozelj, P.; Prots, Yu.; Baitinger, M.; Schwarz, U.; Jung, W. A Borosilicide with Clathrate VIII Structure. *J. Am. Chem. Soc.* **2022**, *144*, 13456-13460.

[5] Palatinus, L.; Brázda, P.; Jelinek, M.; Hrdá, J.; Steciuk, G.; Klementová, M. Specifics of the data processing of precession electron diffraction tomography data and their implementation in the program PETS2.0. *Acta Crystallogr.* **2019**, *B75*, 512-522.

[6] Petříček, V.; Palatinus, L.; Plášil, J.; Dušek, M. Jana2020 – a new version of the crystallographic computing system Jana. *Z. Kristallogr.* **2023**, *238*, 271-282.

[7] Petříček, V.; Dušek, M.; Palatinus, L. Crystallographic computing system Jana2006: general features. *Z. Kristallogr.* **2014**, *229*, 345–352.

[8] Akselrud, L.; Grin, Yu. *J. Appl. Cryst.* **2014**, *47*, 803-805.
